# Effectiveness and Safety of Anti-CD19 Chimeric Antigen Receptor-T Cell Immunotherapy in Patients With Relapsed/Refractory Large B-Cell Lymphoma: A Systematic Review and Meta-Analysis

**DOI:** 10.3389/fphar.2022.834113

**Published:** 2022-04-25

**Authors:** Zhitao Ying, Yuqin Song, Jun Zhu

**Affiliations:** Department of Lymphoma, Key Laboratory of Carcinogenesis and Translational Research (Ministry of Education/ Beijing), Peking University Cancer Hospital & Institute, Beijing, China

**Keywords:** chimeric antigen receptor T cells, CD19, diffuse large B-cell lymphoma, clinical efficacy, meta-analysis

## Abstract

**Aim:** To investigate the effectiveness and safety of using chimeric antigen receptor (CAR) T cell therapies targeting CD19 in patients with diffuse large B-cell lymphoma (DLBCL).

**Methods:** PubMed, Embase, and the Cochrane Library were searched for reports published from database inception up to July 2021. The present meta-analysis included clinical response outcomes, survival outcomes, and safety analyses. For qualitative analysis that could not be combined, the data were presented in a tabular form. Subgroup analyses were also performed according to the costimulatory domains, generic names, and study designs.

**Results:** Twenty-seven studies (1,687 patients) were included. The pooled 12-months overall survival (OS) rate was 63% (95%CI: 56–70%). The pooled best overall response (BOR) was 74.0% (95%CI: 67–79%), with a best complete response (BCR) of 48% (95%CI: 42–54%) and a 3-months CR rate (CRR) of 41% (95%CI: 35–47%). The subgroup analyses by costimulatory domain suggested statistically significant differences in BOR and BCR, whereas not in the 12-months OS rate and 3-months CRR. Among the patients evaluable for safety, 78% (95%CI: 68–87%), 6% (95%CI: 3–10%), 41% (95%CI: 31–52%), and 16% (95%CI: 10–24%) experienced cytokine release syndrome (CRS), severe CRS, neurotoxicity, and severe neurotoxicity, respectively. Compared with the CD28 costimulatory domain, the 4-1BB-based products showed a better safety profile on any-grade CRS (*p* < 0.01), severe CRS (*p* = 0.04), any-grade neurotoxicity (*p* < 0.01), and severe neurotoxicity (*p* < 0.01).

**Conclusion:** Anti-CD19 CAR-T cell immunotherapy has promising effectiveness and tolerable severe AE profile in DLBCL patients. 4-1BB-based CAR-T cells have a similar 12-months OS rate and 3-months CRR with CD28-based products but a better safety profile. The costimulatory domain might not affect the survival outcomes.

## Introduction

Diffuse large B-cell lymphoma (DLBCL) is a clinically heterogeneous class of B cell lymphoma that typically presents as an aggressive or advanced disease but can be curable, even in advanced cases (Martelli et al., 2013; Sehn and Gascoyne, 2015). DLBCL is the most common non-Hodgkin lymphoma (NHL) and represents about 30% of NHL, and its incidence is rising globally (Kuo et al., 2016). This incidence was higher in Pakistan, with 76.4% cases of DLBCL cases detected in 780 samples obtained over 5 years from adult NHL cases (Abid et al., 2005). The crude incidence of DLBCL in Europe is 3.8 per 100,000 persons-year (Tilly et al., 2015) and is increasing with age (from 0.3 per 100,000 persons aged 35–39 years to 26.6 per 100,000 persons aged 80–84 years) (Tilly et al., 2012). About 33% of patients have systemic symptoms at presentation, including constitutional B symptoms (Martelli et al., 2013).

Most patients with DLBCL achieve a good response to first-line rituximab-containing chemoimmunotherapy (Martelli et al., 2013; Sehn and Gascoyne, 2015), but 10–15% of DLBCL patients remain challenging to treat, and 20–35% relapse after an initial response (Sarkozy and Sehn, 2018). The prognosis of such refractory/relapsing (r/r) patients is poor (Sarkozy and Sehn, 2018), with a median overall survival (OS) of 4.4 months and 1- and 2-years OS rates of 23 and 16%, respectively (Van Den Neste et al., 2016). The traditional treatment strategies have poor efficacy in these patients, leading to a short survival (2021).

One of the latest developments in cell immunotherapy is chimeric antigen receptor T (CAR-T) cells, demonstrating remarkable achievements in B-cell NHL (Kersten et al., 2020; Yin et al., 2021). CAR-T cell therapy involves autologous or allogeneic genetically engineered T cells to fight cancer and appears to be effective even in r/r patients (Kersten et al., 2020; Yin et al., 2021). Indeed, CAR-T cells engineered to target CD19 have shown remarkable efficacy in patients with r/r CD19^+^ B-cell malignancies (Lee et al., 2015; Turtle et al., 2016; Locke et al., 2017; Kersten et al., 2020).

As of April 2021, four CAR-T cell therapies have been approved by the US Food and Drug Administration (FDA) for CD19-expressing hematologic malignant cancers: tisagenlecleucel, axicabtagene ciloleucel, brexucabtagene autoleucel, and lisocabtagene maraleucel. In addition to this, the National Medical Products Administration (NMPA) of China has approved two products of relmacabtagene autoleucel and axicabtagene ciloleucel for the treatment of adult patients with relapsed or refractory large B-cell lymphoma (r/r LBCL) after two or more lines of systemic therapy up to now. Still, there is heterogeneity in the manufacturing technologies among the available CAR-T cell therapies, including the costimulatory domain. In addition, many other CAR-T cell therapies are under development, using different manufacturing processes (Feins et al., 2019; Subklewe et al., 2019; Rafiq et al., 2020; Zhang et al., 2020; Sterner and Sterner, 2021). Furthermore, the available studies vary in patient populations, CAR-T cell constructs, gene transfer methods, infused CAR-T cell doses, preconditioning chemotherapy, persistence of CAR-T cells after treatment, peak cytokine levels, and treatment toxicity. Furthermore, how the differences in the manufacturing technologies contribute to CAR-T cell therapy efficacy and safety, especially OS in DLBCL patients, remains unclear.

Therefore, this systematic review and meta-analysis included clinical trials and observational studies to investigate the effectiveness and safety of using CAR-T cells targeting CD19 in patients with DLBCL.

## Methods

### Literature Search

This systematic review and meta-analysis was conducted in conformity with the Preferred Reporting Items for Systematic Reviews and Meta-Analyses (PRISMA) guidelines (Moher et al., 2009). The relevant articles were searched according to the PICOS principle, followed by screening based on the inclusion and exclusion criteria. This study was registered with PROSPERO (CRD42021274526).

The inclusion criteria were 1) patients (aged ≥18 years old) with measurable, histologically confirmed r/r DLBCL, including the subtypes based on the 2008 WHO Classification, and who failed to at least two lines of systemic treatment; 2) interventions: anti-CD19 CAR-T cell immunotherapy; 3) outcome: any studies reporting the overall response rate (ORR), or complete response (CR), or cytokine release syndrome (CRS), or neurotoxicity; 4) design: clinical trials or observational studies; 5) publication type: full-text or conference abstract; 6) published in English.

The exclusion criteria were 1) report with incomplete or inconsistent original data, too little information, or lack of data; 2) duplicated publications (in which case only the most recent one was included); 3) without efficacy evaluation for DLBCL patients after CAR-T treatment; 4) case report; 5) non-human studies; 6) less than 10 patients.

PubMed, Embase, and the Cochrane Library were searched for reports published from database inception up to July 2021 using the MeSH terms of “Chimeric Antigen Receptor T Cells”, “CD19”, and ‘B cell lymphoma”, as well as relevant keywords. The search was performed independently by two investigators (Z. Ying and Y. Song). Discrepancies in the search were solved by a discussion with a third investigator (J. Zhu).

### Data Extraction

Study characteristics (author, publication year, study type, country where the study was performed, and study design), patients’ characteristics (sex, sample size, age, and histological type), CAR-T cell type, and manufacturing process (T-cell origine, vector, dose, and co-stimulation domain), clinical response outcomes (best overall response (BOR), best complete response (BCR), 3-months complete response rate (CRR)), survival outcomes (12-months OS rate, median OS, median progression-free survival (PFS), and median duration of response (DOR), and safety outcomes (any CRS events, severe CRS events, any neurotoxicity events, and severe neurotoxicity events) were extracted by two different investigators (Z Ying and Y Song), independently, using a standardized form. Differences in data extraction were solved by discussion among Z. Ying, Y. Song and J. Zhu until a consensus was reached.

### Quality of the Evidence

The level of evidence of the included articles was assessed independently by two investigators (Yuqin Song and Jun Zhu) according to the Methodological Index for Non-Randomized Studies (MINORS) tool for clinical trials and the Newcastle-Ottawa Scale (NOS) tool for observational studies (Slim et al., 2003). Discrepancies in the assessment were resolved through discussion among Z. Ying, Y. Song and J. Zhu until a consensus was reached.

### Statistical Analysis

The statistical analysis was conducted using R version 4.1.0. Because of the diversity among the included studies, the random-effects model was used regardless of heterogeneity. Heterogeneity was determined using forest plots and the I^2^ index. Results were reported as proportions with 95% confidence intervals (CIs). For qualitative analysis such as median OS, median PFS, and median DOR, the data were presented in a tabular form. The subgroup analyses were preplanned and were performed according to study design, generic name, and costimulatory domain. The potential publication bias was evaluated using funnel plots.

## Results

### Study Characteristics and Quality Assessment

**FIGURE 1:**
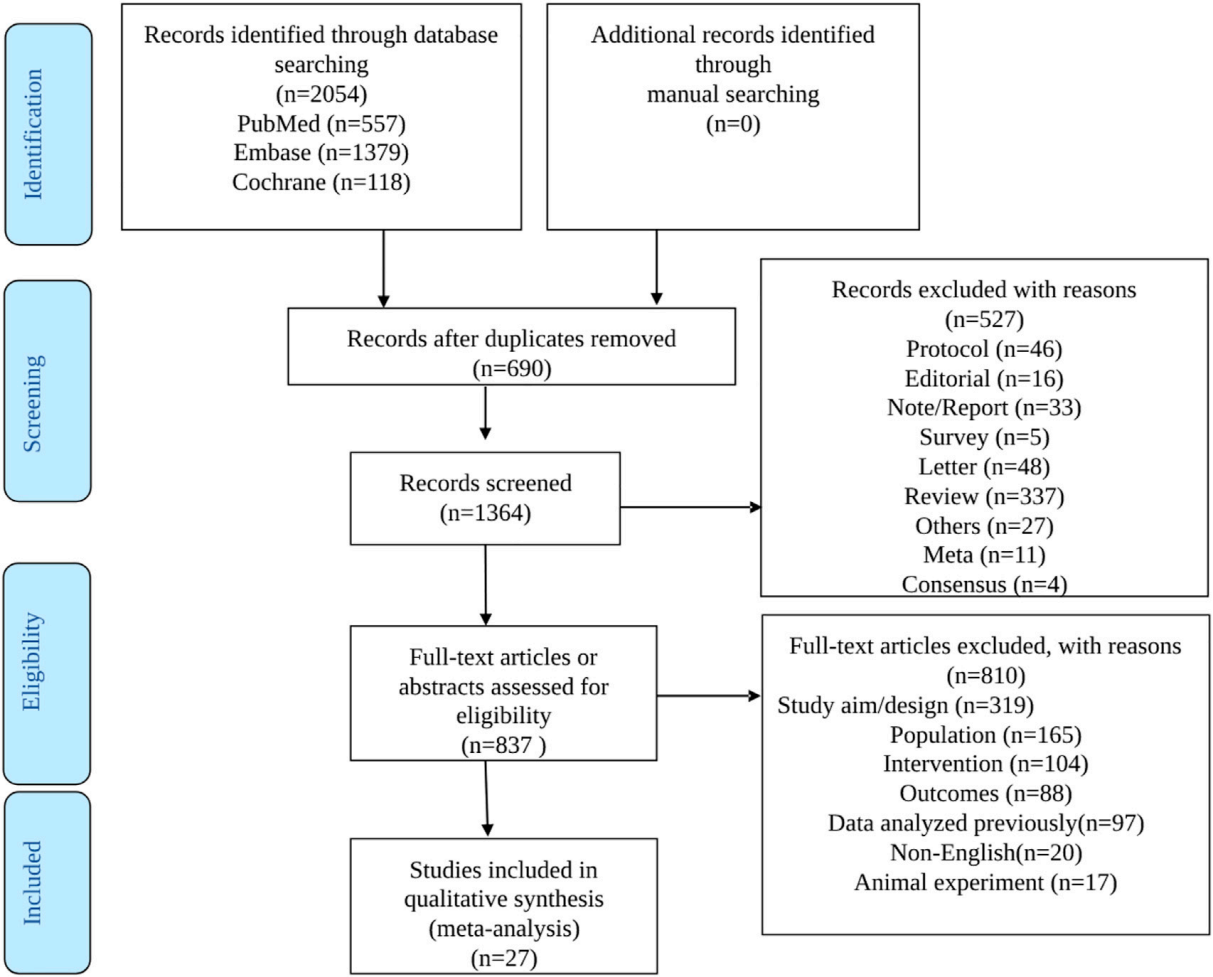
Study selection process diagram.

The literature search process is illustrated in Figure 1. The initial search yielded 2054 records. Then, among the 1,364 left after removing the duplicates, 527 records were excluded, and 837 full-text articles were assessed for eligibility. After excluding 810 studies (319 for study aim or design, 165 for the population, 104 for the intervention, 88 for the outcomes, 97 for reporting previously reported data, 20 for non-English full text, and 17 for being *in vivo* studies), 13 full-text articles and 14 conference abstracts were included.

The characteristics of the 27 included studies are presented in Table 1. The studies included a total of 1,687 patients with DLBCL. Sixteen studies were from the United States, five from Europe, four from China, and two were multicenter studies performed in multiple countries. Eight studies reported data about tisagenlecleucel, fourteen about axicabtagene ciloleucel, one about relmacabtagene autoleucel, two about lisocabtagene maraleucel, and four about non-commercial preparations.

**TABLE 1:** Baseline characteristics of included studies.

Author	Study Design	Registration number	Start of data	Country	Sample Size	Age, years (Mean/Median)	Sex, % (male)	Histological type, n	CAR T	Mode of Transduction	Co-stimulatory Domain	Dose	Original T Cell Sources	Lymphodepletion	Does
Ayuk (2020)	Clinical trial, CA	—	—	Germany	18	—	—	DLBCL 18	axi-cel	retroviral vector	CD28	—	autologous	—	—
Jacobson et al. (2019)	ZUMA-6, CA	NCT02926833	Sep-16	United States of America	12	55 (30–66)	—	DLBCL 12	axi-cel	retroviral vector	CD28	2 × 10⁶	autologous	—	—
(Cohort 1) Jacobson et al. (2020)	ZUMA-9, CA	NCT03153462	—	United States of America	25	56 (28–76)	60	DLBCL 20	axi-cel	retroviral vector	CD28	2 × 10⁶	autologous	fludarabine + cyclophosphamide	fludarabine: 30 mg/m^2^/day for 3 days; cyclophosphamide: 500 mg/m^2^/day for 3 days
Locke et al. (2019)	ZUMA-1	NCT02348216	Jan-15	USA/Israel	111	58 (23–76)	67	PMBCL 8/TFL 16	axi-cel	retroviral vector	CD28	2 × 10⁶	autologous	fludarabine + cyclophosphamide	fludarabine: 30 mg/m^2^ body-surface area per day, days −5, −4, and −3; cyclophosphamide: 500 mg/m^2^ body-surface area per day, days −5, −4, and −3
Huang et al. (2020)	Clinical trial	NCT01865617	May-13	China	11	49 (29–69)	72.7	DLBCL 9	lab	lentiviral vector	4-1BB	1.8–3×10^6^	autologous	fludarabine + cyclophosphamide	fludarabine: 25 mg/m^2^/d (d1–3),cyclophosphamide: 900 mg/m^2^/d (d3–4)
Kochenderfer et al. (2017)	Clinical trial	NCT00924326	Feb-09	France	19	—	—	DLBCL-NOS 13/TFL 4/PMBCL 2	lab	retroviral vector	CD28	1 × 10⁶, 2 × 10⁶, 6 × 10⁶	autologous	fludarabine + cyclophosphamide	Fludarabine:30 mg/m^2,^ Days -5 to -3; cyclophosphamide: 300 or 500 mg/m^2^, Days -5 to -3
Yan et al. (2019)	Clinical trial	NCT03355859	Jan-18	China	10	47 (32–59)	80	GCBC 2/Non-GCBC 7/MZBCL 1	lab	lentiviral vector	4-1BB	2.5 × 10^7^, 5.0 × 10^7^, 1.0 × 10^8^	autologous	fludarabine + cyclophosphamide	fludarabine: 25 mg/m^2^/day; cyclophosphamide: 250 mg/m^2^/day on Day -4 to Day -2
Zhou et al. (2020)	Clinical trial	ChiCTR-OOC-16007,779	May-16	China	21	—	62	DLBCL 12/High-grade BCL 1/PMLBCL 1	lab	lentiviral vector	CD28, CD27 (4th generation)	median dose, 8.9×10^5^ (0.3×10^5^–48.0×10^5^)	autologous	fludarabine + cyclophosphamide	fludarabine: 25 mg/m^2^ for 3 days; cyclophosphamide: 900 mg/m^2^ for 1 day
Abramson et al. (2020)	TRANSCEND NHL 001	NCT 02631044	Jan-16	United States of America	269	63 (18–86)	65	DLBCL-NOS 137/TFL 60/PMBCL 15/DHL THL 36/Grade 3B GFL 3	liso-cel	lentiviral vector	4-1BB	50 × 10⁶, 100 × 10⁶, 150 × 10⁶	autologous	fludarabine + cyclophosphamide	fludarabine: 30 mg/m^2^ daily for 3 days; cyclophosphamide: 300 mg/m^2^ daily for 3 days
Godwin et al. (202)	OUTREACH, CA	NCT03744676	Nov-18	United States of America	46	63 (34–83)	—	DLBCL-NOS 29	liso-cel	lentiviral vector	4-1BB	—	autologous	—	—
Ying et al. (2021)	RELIANCE	NCT04089215	Jun-19	China	59	56 (18–75)	55.9	DLBCL-NOS 41/TFL 9/PMBCL 4/Grade 3B FL 2/DHL THL 3	relma-cel	lentiviral vector	4-1BB	100 × 10⁶, 150 × 10⁶	autologous	fludarabine + cyclophosphamide	fludarabine: 25 mg/m^2^ i.v. daily, for 3 days; cyclophosphamide: 250 mg/m^2^ i.v. daily, for 3 days
Chavez et al. (2020)	clinical trial, CA		—	United States of America	10	arm 1: 59 (32–67); arm 2: 64 (58–76)	—	ABC 3, GCB 3	tisa-cel	lentiviral vector	4-1BB	0.6–6.0×10^8^	autologous	fludarabine + cyclophosphamide/bendamustine	1. fludarabine (25 mg/m2) and cyclophosphamide (250 mg/m2) daily for 3 days 2. bendamustine (90 mg/m2) daily for 2 days
Schuster et al. (2019)	Juliet	NCT 02445248	Jul-15	USA/Australia/Canada/France/Germany/Italy/Japan/Nertherlands/Norway	111	56 (22–76)	—	DLBCL-NOS 88/TFL 21/DHL 19/THL 70	tisa-cel	lentiviral vector	4-1BB	median dose, 3.0×10^8^ (0.1×10^8^–6.0×10^8^	autologous	fludarabine + cyclophosphamide/bendamustine	1. fludarabine (25 mg/m2) and cyclophosphamide (250 mg/m2) daily for 3 days 2. bendamustine (90 mg/m2) daily for 2 days
Nastoupil et al. (2020)	RWS	—	Sep-18	United States of America	298	60 (21–83)	64	DLBCL 203/PMBCL 19/TFL 76	axi-cel	retroviral vector	CD28	—	autologous	fludarabine + cyclophosphamide	fludarabine: 30 mg/m^2^ body-surface area per day, days −5, −4, and −3; cyclophosphamide: 500 mg/m^2^ body-surface area per day, days −5, −4, and −3
Sesques et al. (2020)	RWS	—	Jan-18	France	61	59 (27–75)	66	DLBCL 38/PMBCL 4/TFL 18/TMZL 1	tisa-cel, axi-cel	retroviral vector, lentiviral vector	CD28, 4-1BB	—	autologous	fludarabine + cyclophosphamide/bendamustine	1. (for axi-cel)fludarabine: 30 mg/m^2^ day 1 to day 3 and cyclophosphamide: 500 mg/m^2^, day -5 to day -3; (for tisa-cel)fludarabine: 30 mg/m2 day 1 to day 3 and cyclophosphamide: 250 mg/m2, day -5 to day -3 2.bendamustine: 90 mg/m^2^ day -5 and day -4
Jaglowski et al. (2019)	RWS, CA	—	—	United States of America	70	65.11 (18.5–88.9)	61.4	high-grade BCL 22/DLBCL 21/GCBC 13/ABC 10	tisa-cel	lentiviral vector	4-1BB	—	autologous	—	—
Iacoboni et al. (2021)	RWS	—	Dec-18	Spain	75	60 (52–67)	59	DLBCL-NOS 44/DH TH 11/TFL 17	tisa-cel	lentiviral vector	4-1BB	3.5 × 10^8^	autologous	fludarabine + cyclophosphamide	fludarabine: 25 mg/m^2^/day, for 3 days; cyclophosphamide: 250 mg/m^2^/day, for 3 days
Faramand et al. (2019)	Prospective,CA	—	—	United States of America	20	64 (43–73)	—	r/r DLBCL 20	axi-cel	retroviral vector	CD28	—	autologous	—	—
Dean et al. (2019)	Retrospective, CA	—	Jun-15	United States of America	48	63 (28–76)	64.6	—	axi-cel	retroviral vector	CD28	—	autologous	—	—
Grana et al. (2021)	Retrospective	—	Jan-18	United States of America	37	59 (23–75)	59.5	DLBCL 22/PMBCL 4/High grade BCL 2/TFL 9	axi-cel	retroviral vector	CD28	—	autologous	—	—
Melody et al. (2019)	Retrospective, CA	—	Jun-18	United States of America	50	53 (26–67)	74	DLBCL 27/PMBCL 5/TFL 8/High grade BCL 7	axi-cel	retroviral vector	CD28	—	autologous	—	—
Mian et al. (2020)	Retrospective, CA	—	May-18		27	63 (48–68)	67	r/r BCL 27	axi-cel	retroviral vector	CD28	—	autologous	—	—
Panaite et al. (2020)	Retrospective, CA	—	Feb-18	United States of America	53	63 (25–79)	68	DLBCL 41/PMBCL 1/TFL 11	axi-cel	retroviral vector	CD28	—	autologous	—	—
Pennisi et al. (2020)	Retrospective	—	Feb-18	United States of America	49	64 (20–85)	69	DLBCL 49	axi-cel, tisa-cel	retroviral vector, lentiviral vector	CD28, 4-1BB	—	autologous	—	—
Strati et al. (2019)	Retrospective, CA	—	Jan-18	United States of America	95	60 (18–85)	71	DLBCL 71/TFL 17/PMLBCL 6	axi-cel	retroviral vector	CD28	—	autologous	—	—
Iacoboni et al. (2020)	Retrospective, CA	—	Dec-18	Spain	45	53 (23–72)	64	r/r DLBCL 45	tisa-cel	lentiviral vector	4-1BB	—	autologous	—	—
Lee et al. (2020)	Retrospective, CA	—	Jan-19	United States of America	37	60, SD 18	65	DLBCL 37	tisa-cel	lentiviral vector	4-1BB	—	autologous	—	—

Abbreviation; DLBCL-NOS, DLBCL, not otherwise specified; PMBCL, primary mediastinal B-cell lymphoma; FL, Follicular lymphoma; DHL/THL, double-/triple-hit lymphoma; liso-cel, Lisocabtagene maraleucel; tisa-cel, Tisagenlecleucel; relma-cel, Relmacabtagene autoleucel; axi-cel, Axicabtagene ciloleucel

The quality assessment of the included studies is presented in Supplementary Table S1 (NOS) and Supplementary Table S2 (MINORS). Among the observational studies, two scored five stars on the NOS, one scored six stars, one scored seven stars, and one scored eight stars. The clinical trials scored 19–24 points on the MINORS.

### Response Rate

**FIGURE 2:**
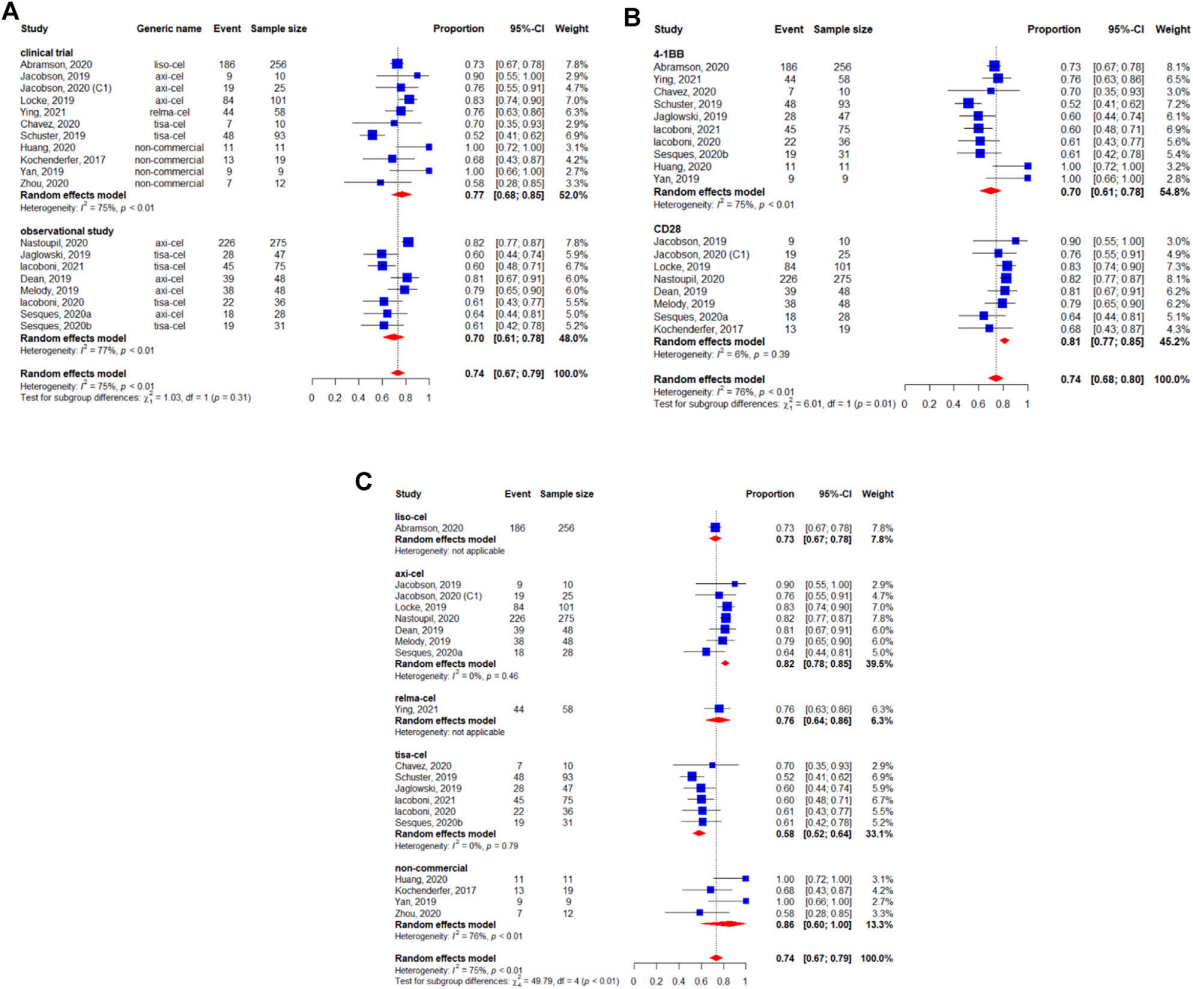
Subgroup analyses of best objective response rate (BOR) of patients with diffuse large B-cell lymphoma (DLBCL) treated with chimeric antigen receptor T cells (CAR-Ts) according to **(A)** study design, **(B)** the costimulatory domain, and **(C)** type of chimeric antigen receptor T cells (CAR-T).

A total of 1,192 patients were evaluable for the BOR. The pooled BOR was 74.0% (95%CI: 67–79%) (Figure 2). Subgroup analyses by study design did not show statistically significant differences (*p* = 0.31) (Figure 2A). The subgroup analysis by costimulatory domain suggested the presence of a significant difference between CD28^−^and 4-1BB-based CAR-T cell therapies (*p* = 0.01) (Figure 2B). Among these CAR-T cell products, the subgroup analysis by generic name showed that the BORs for tisagenlecleucel, axicabtagene ciloleucel, lisocabtagene maraleucel, and relmacabtagene autoleucel were 58% (95%CI: 52–64%), 82% (95%CI: 78–85%), 73% (95%CI: 67–78%), and 76% (95%CI: 64–86%), respectively, and 86% (95%CI: 60–100%) for non-commercial CAR-T cell products (Figure 2C).

**FIGURE 3:**
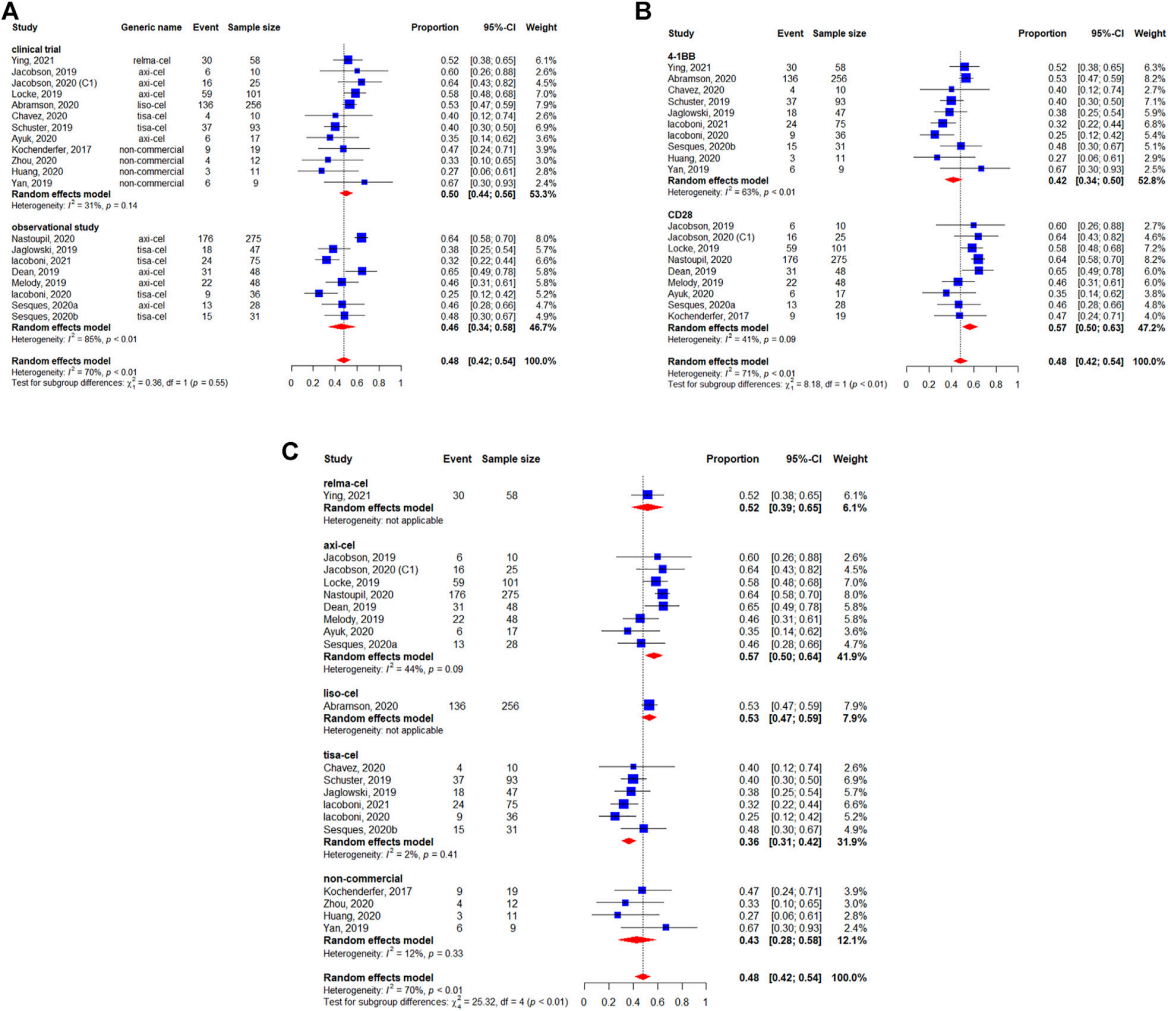
Subgroup analyses of best complete response (BCR) rate of patients with diffuse large B-cell lymphoma (DLBCL) treated with chimeric antigen receptor T cells (CAR-Ts) according to **(A)** study design, **(B)** the costimulatory domain, and **(C)** type of chimeric antigen receptor T cells (CAR-T).

The BCR was reported for 1,209 patients. The pooled BCR was 48% (95%CI: 42–54%) (Figure 3). The study design was not related to a better BCR (*p* = 0.55) (Figure 3A). However, we observed a statistically significant difference in the subgroup analysis by the costimulatory domain (*p* < 0.01) (Figure 3B). Regarding the subgroup analysis by generic name, the results suggested a difference between each CAR-T product. The BCR was 53% (95%CI: 47–59%) for lisocabtagene maraleucel, 57% (95%CI: 50–64%) for axicabtagene ciloleucel, 52% (95%CI: 39–65%) for relmacabtagene autoleucel, 36% (95%CI: 31–42%) for tisagenlecleucel, and 43% (95%CI: 28–58%) for non-commercial products (Figure 3C).

**FIGURE 4:**
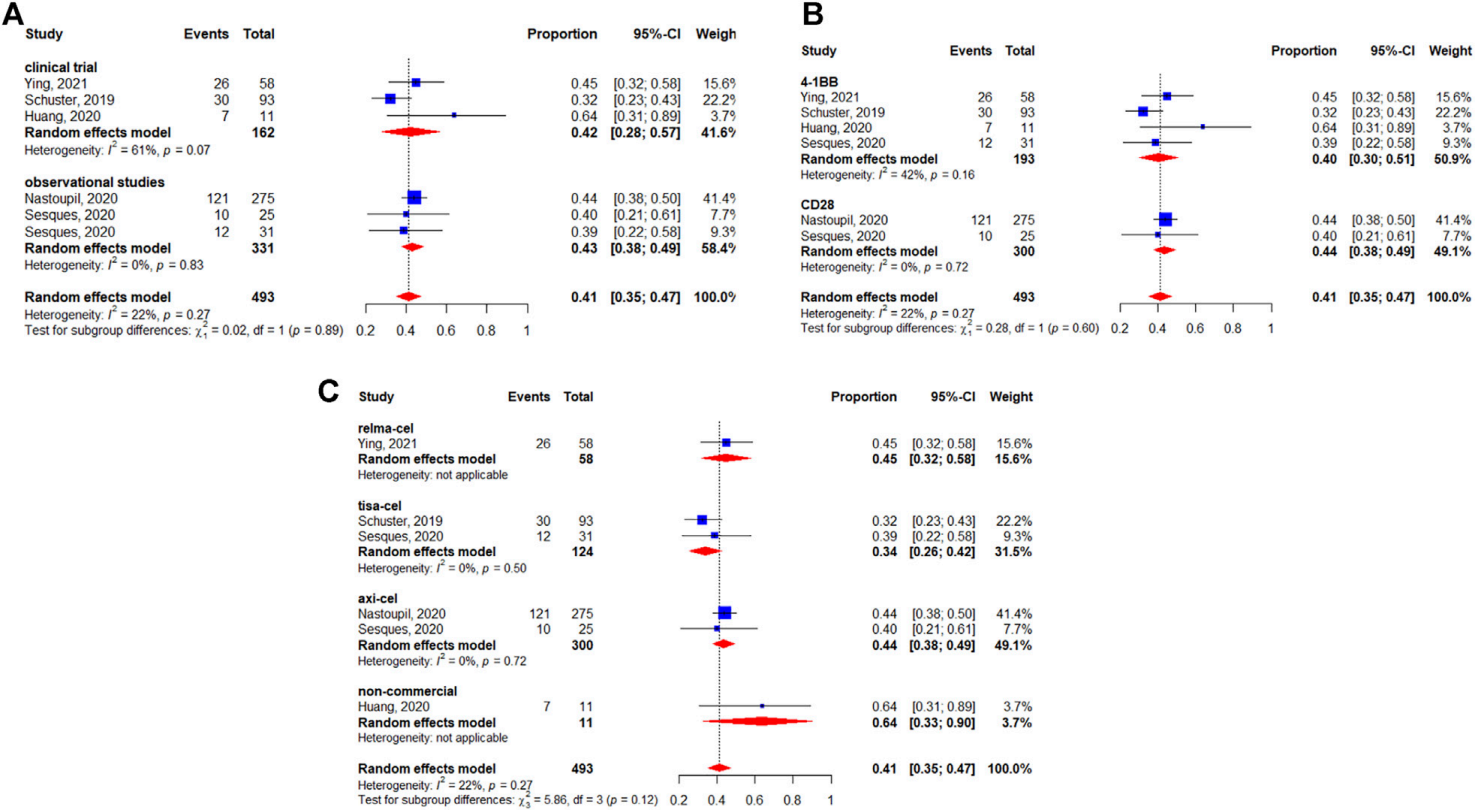
Subgroup analyses of 3-months complete response (CR) rate of patients with diffuse large B-cell lymphoma (DLBCL) treated with chimeric antigen receptor T cells (CAR-Ts) according to **(A)** study design, **(B)** the costimulatory domain, and **(C)** type of chimeric antigen receptor T cells (CAR-T).

The 3-months CRR was evaluable in 493 patients, and the pooled 3-months CRR was 41% (95%CI: 35–47%) (Figure 4). The subgroup analyses suggested that the 3-months CRRs were similar between study designs (*p* = 0.89) (Figure 4A), costimulatory domains (*p* = 0.60) (Figure 4B), and generic names (*p* = 0.12) (Figure 4C).

### Survival Outcome

**FIGURE 5:**
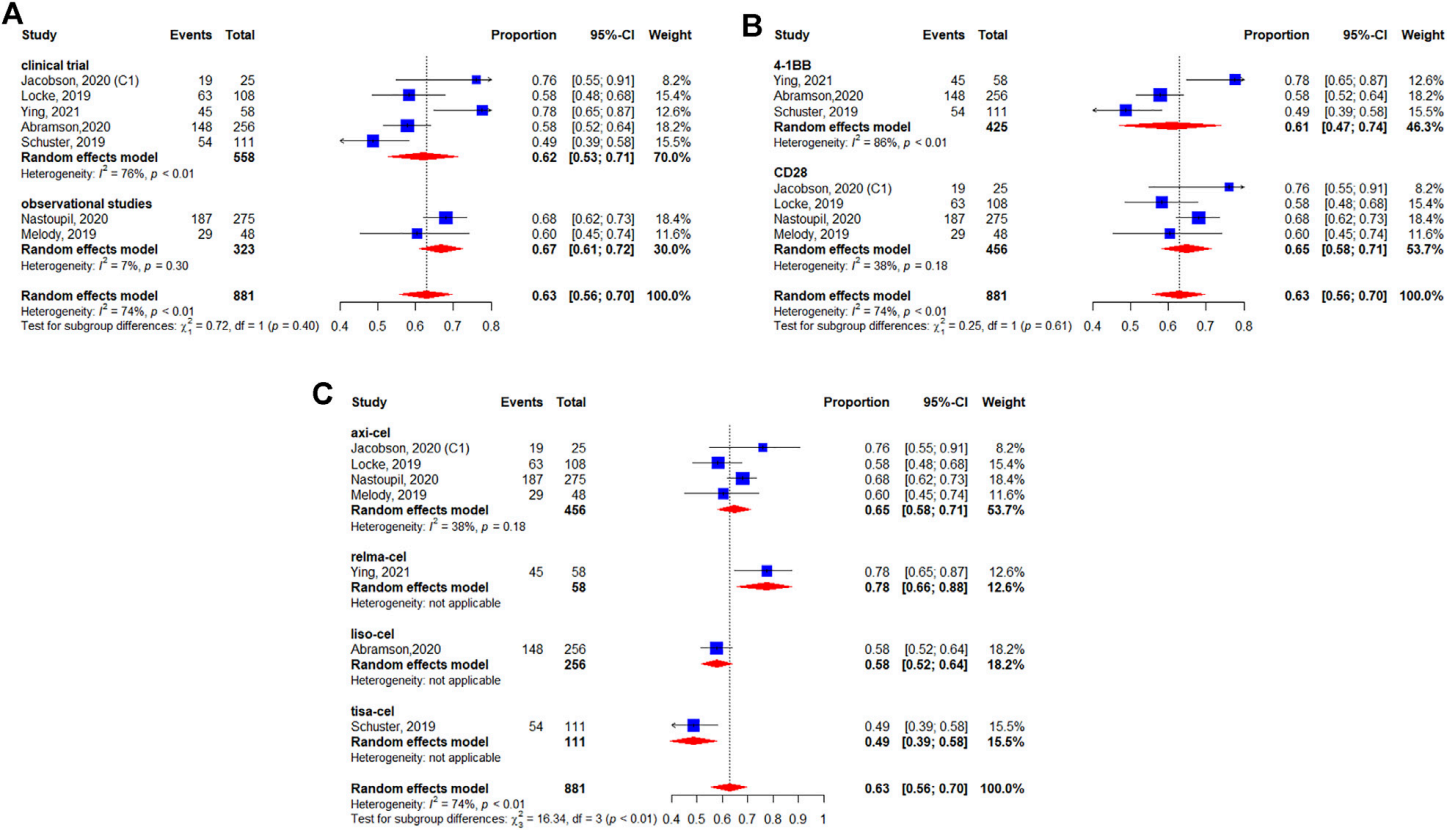
Subgroup analyses of 12-months OS rate by type of chimeric antigen receptor T cells (CAR-T) **(A)** study design, **(B)** the costimulatory domain, and **(C)** type of chimeric antigen receptor T cells (CAR-T).

Five studies reported the median OS (Schuster et al., 2019; Abramson et al., 2020; Jacobson et al., 2020; Zhou et al., 2020; Iacoboni et al., 2021). The median survival varied from 12.0 (95%CI: 7.0-Not reached) months to 27.3 (95%CI: 16.2–45.6) months. The longest median OS was 27.3 (95%CI: 16.2–45.6) months with 29.3 months of median follow-up in patients treated with lisocabtagene maraleucel (Table 2). The 12-months OS rate was 63% (95%CI: 56–70%) (Figure 5). The subgroup analyses indicated that the 12-months OS rates were similar between study designs (*p* = 0.40) (Figure 5A) and costimulatory domains (*p* = 0.61) (Figure 5B). The 12-months OS rates with axicabtagene ciloleucel, relmacabtagene autoleucel, lisocabtagene maraleucel, and tisagenlecleucel were 65% (95%CI: 58–71%), 78% (95%CI: 66–88%), 58% (95%CI: 52–64%), and 49% (95%CI: 39–58%), respectively (Figure 5C).

**TABLE 2:** Survival outcome of OS.

Study	CAR T	Median Overall Survival, months	Median Follow up, months
Jacobson et al. (2020)	axi-cel	23.8 (13.5–NR)	27.1
Abramson et al. (2020)	liso-cel	27.03 (16.2–45.6)	29.3
Schuster et al. (2019)	tisa-cel	12.0 (7.0–NR)	14.0
Zhou et al. (2020)	lab	23.8	13.7
Iacoboni et al. (2021)	tisa-cel	10.7 (7.4–NR)	14.1

CAR T: chimeric antigen receptor T cells; NR: not reached

Six studies reported the median PFS (Locke et al., 2019; Abramson et al., 2020; Nastoupil et al., 2020; Grana et al., 2021; Iacoboni et al., 2021; Ying et al., 2021). The median PFS varied from 3.0 (95%CI: 2.6–4.7) to 8.3 (95%CI: 6.0–15.1) months. The longest median PFS was 8.3 (95%CI: 6.0–15.1) months at a median follow-up of 12.9 months in patients treated with axicabtagene ciloleucel (Table 3).

**TABLE 3:** Survival outcome of PFS.

Study	CAR T	Median Progression-free Survival, months	Median Follow-Up, months
Locke et al. (2019)	axi-cel	5.9 (3.3–15.0)	27.1
Abramson et al. (2020)	liso-cel	6.8 (3.3–12.7)	23.9
Ying et al. (2021)	relma-cel	7.0 (4.8-NR)	17.9
Nastoupil et al. (2020)	axi-cel	8.3 (6.0–15.1)	12.9
Iacoboni et al. (2021)	tisa-cel	3.0 (2.6–4.7)	14.1
Grana et al. (2021)	axi-cel	5.8	6.0

CAR T: chimeric antigen receptor T cells; NR: not reached.

The median DOR was reported in five studies (Locke et al., 2019; Abramson et al., 2020; Zhou et al., 2020; Iacoboni et al., 2021; Ying et al., 2021). The median DOR varied from 6.8 to 23.1 months. The longest median DOR was 23.1 (95%CI: 8.6-NR) months at a median follow-up of 23 months in patients treated with lisocabtagene maraleucel (Table 4).

**TABLE 4:** Clinical outcome of the duration of response (DOR).

Study	CAR T	Median Duration of Response, months	Median Follow up, months
Locke et al. (2019)	axi-cel	11.1 (4.2-NE)	27.1
Ying et al. (2021)	relma-cel	8.0 (6.0-NR)	8.9
Zhou et al. (2020)	lab	6.8	13.7
Iacoboni et al. (2021)	tisa-cel	8.9 (2.2-NE)	14.1
Abramson et al. (2020)	Liso-cel	23.1 (8.6-NR)	23.0

CAR T: chimeric antigen receptor T cells; NE: not estimated; NR: not reached.

### Safety Analyses

**FIGURE 6:**
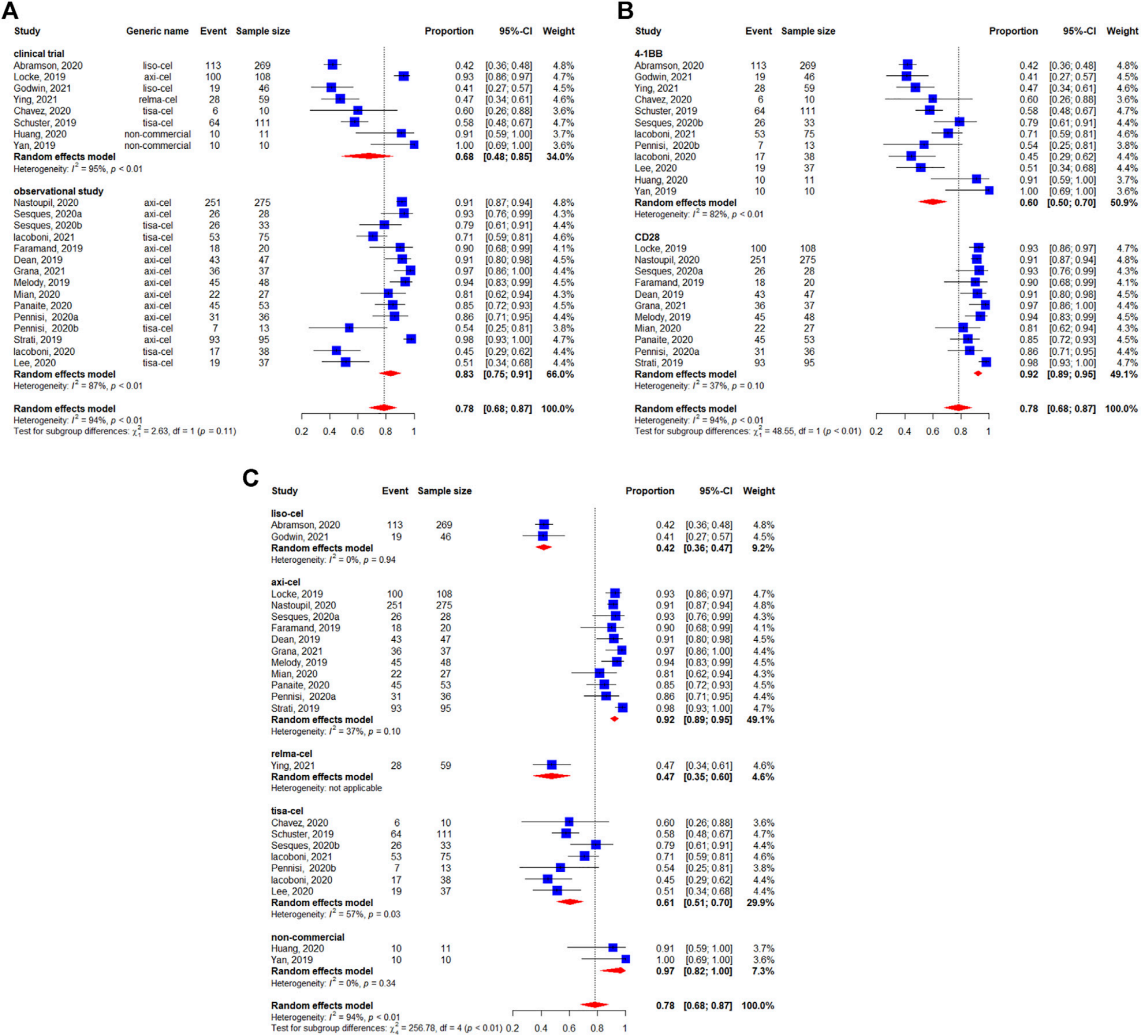
Any**-**grade cytokine release syndrome (CRS) of patients with diffuse large B-cell lymphoma (DLBCL) treated with chimeric antigen receptor T cells (CAR-Ts) according to **(A)** study design, **(B)** the costimulatory domain, and **(C)** type of chimeric antigen receptor T cells (CAR-T).

Among the 1,486 patients evaluable for safety, 78% (95%CI: 68–87%) experienced any-grade CRS (Figure 6). In the subgroup analyses, we didn’t observe the statistically significant difference regarding the study design (*p* = 0.11) (Figure 6A). Figure 6B shows that the occurrence of any-grade CRS was higher for the CD28 CAR-T cell products (92%, 95%CI: 89–95%) than for the 4-1BB CAR-T cell products (60%, 95%CI: 50–70%) (*p* < 0.01). On the other hand, considerable differences could be observed in the CRS rates among the different preparations, with 92% (95%CI: 89–95%) for axicabtagene ciloleucel, 42% (95%CI: 36–47%) for lisocabtagene maraleucel, 47% (95%CI: 35–60%) for relmacabtagene autoleucel, 61% (95%CI: 51–70%) for tisagenlecleucel, and 97% (95%CI: 82–100%) for non-commercial preparations (Figure 6C).

**FIGURE 7:**
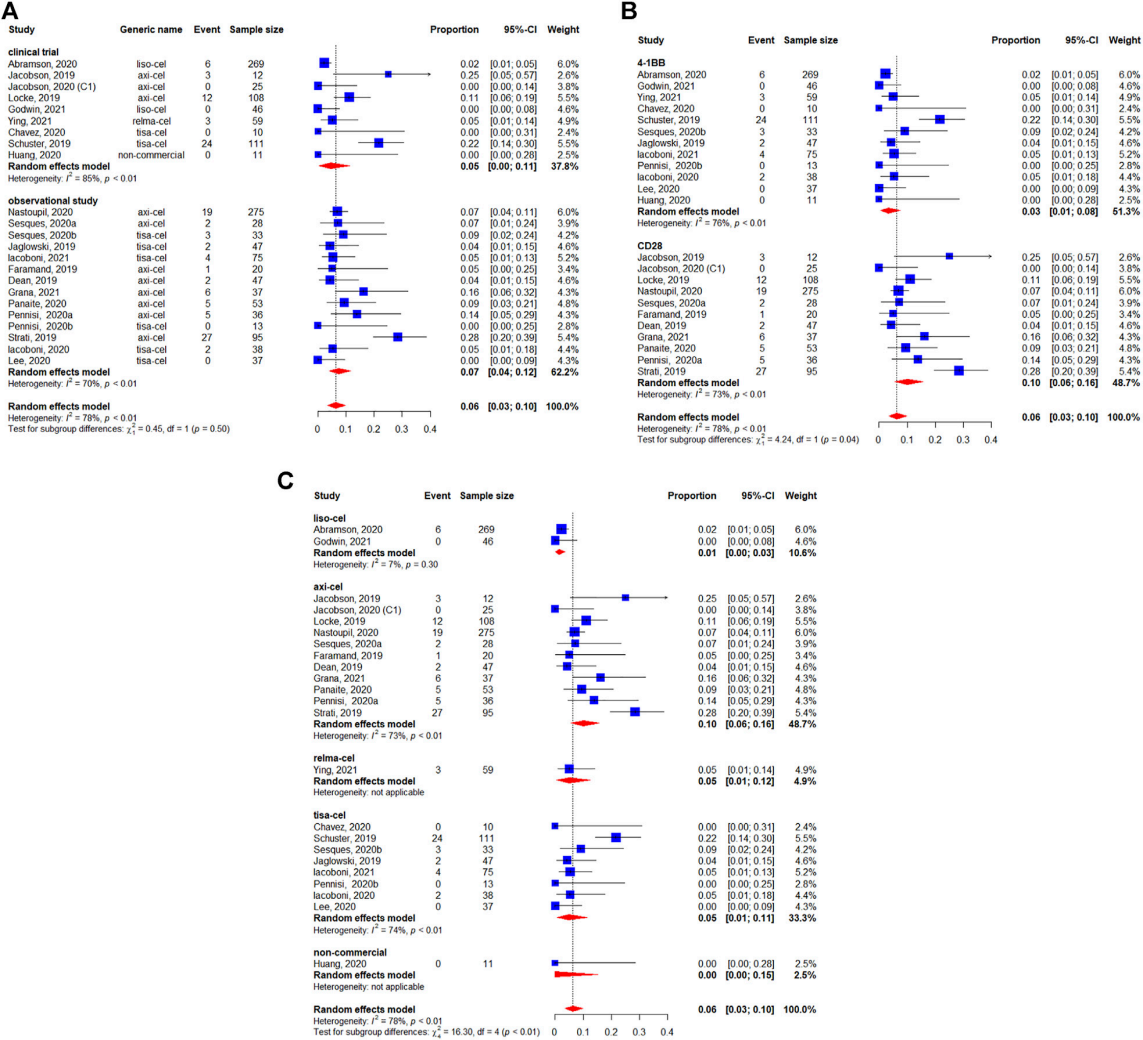
Severe (grade ≥3) cytokine release syndrome (CRS) of patients with diffuse large B-cell lymphoma (DLBCL) treated with chimeric antigen receptor T cells (CAR-Ts) according to **(A)** study design, **(B)** the costimulatory domain, and **(C)** type of chimeric antigen receptor T cells (CAR-T).

Severe CRS (i.e., grade ≥3) occurred in 6% of 1,485 evaluable patients (95%CI: 3–10%) (Figure 7). There were no apparent differences between study designs (*p* = 0.50) (Figure 7A), whereas the differences was observed between costimulatory domains (*p* = 0.04) (Figure 7B). The occurrence of grade ≥3 CRS rates were 10, 1, 5, 5, and 0% for axicabtagene ciloleucel, lisocabtagene maraleucel, relmacabtagene autoleucel, tisagenlecleucel, and non-commercial products, respectively (Figure 7C).

**FIGURE 8:**
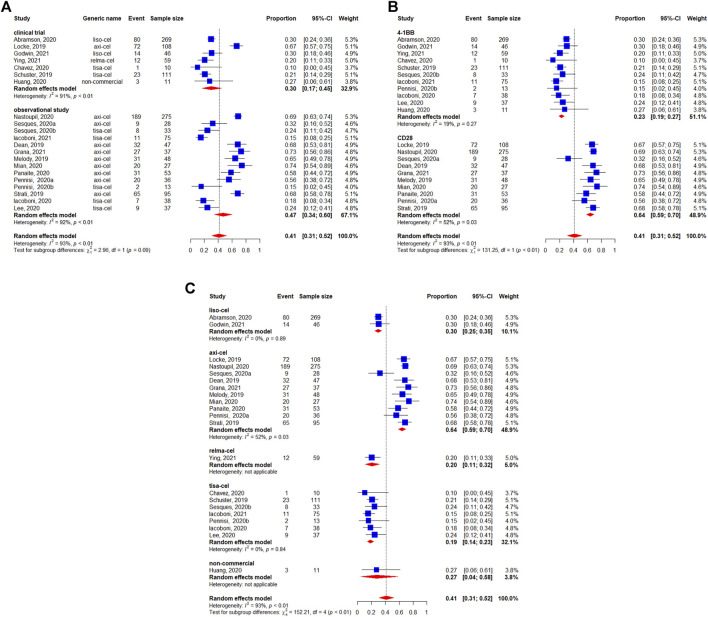
Any-grade neurotoxicity of patients with diffuse large B-cell lymphoma (DLBCL) treated with chimeric antigen receptor T cells (CAR-Ts) according to **(A)** study design, **(B)** the costimulatory domain, and **(C)** type of chimeric antigen receptor T cells (CAR-T).

The pooled incidence of any-grade neurotoxicity was 41% (95%CI: 31–52%) among 1,456 patients, with a considerable variation among the included studies (Figure 8). No differences was observed in the subgroup analyses of study design regarding incidence of any-grade neurotoxicity events (*p* = 0.09) (Figure 8A). The statistically significant differences were observed between the co-stimulatory domains, with 23% (95%CI: 19–27%) for 4-1BB and 64% (95%CI: 59–70%) for CD28 (*p* < 0.01) (Figure 8B). The neurotoxicity rates were 64% (95%CI: 59–70%) for axicabtagene ciloleucel, 30% (95%CI: 25–35%) for lisocabtagene maraleucel, 20% (95%CI: 11–32%) for relmacabtagene autoleucel, 19% (95%CI: 14–23% for tisagenlecleucel, and 27% (95%CI: 4–58%) for non-commercial products (Figure 8C).

**FIGURE 9:**
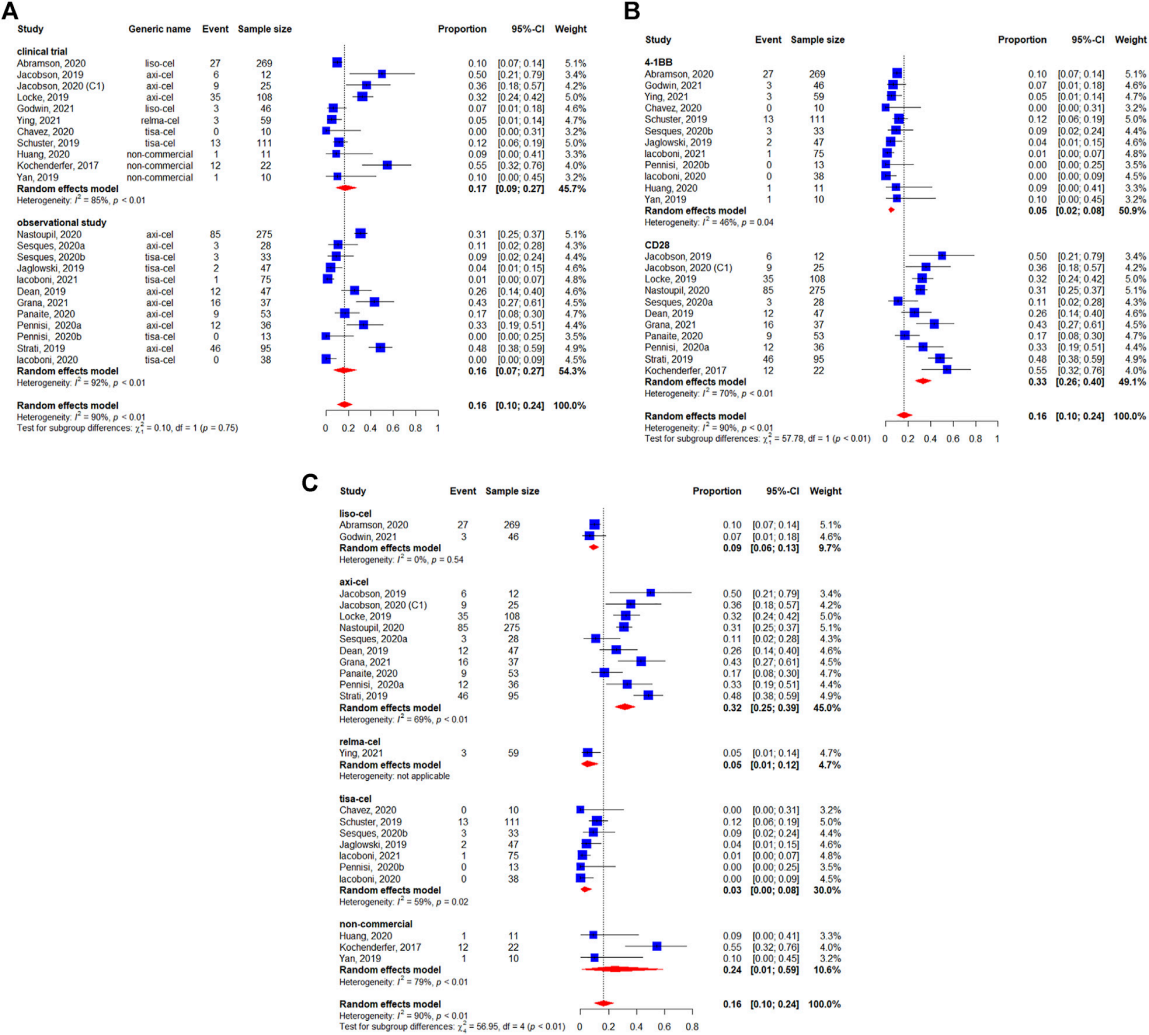
Severe (grade ≥3) neurotoxicity of patients with diffuse large B-cell lymphoma (DLBCL) treated with chimeric antigen receptor T cells (CAR-Ts) according to **(A)** study design, **(B)** the costimulatory domain, and **(C)** type of chimeric antigen receptor T cells (CAR-T).

Severe neurotoxicity (grade ≥3) occurred in 16% (95%CI: 10–24%) of 1,460 patients (Figure 9). We did not observe statistically significant differences between study designs (*p* = 0.75) (Figure 9A). The subgroup analysis by co-stimulatory domain suggested a lower incidence of severe neurotoxicity for 4-1BB-based CAR-T cells [5% (95%CI: 2–8%)] compared with CD28-based CAR-T cells [33% (95%CI: 26–40%)] (*p* < 0.01) (Figure 9B). Finally, the incidence of severe neurotoxicity appeared to be higher with axicabtagene ciloleucel [32% (95%I: 25–39%)] and non-commercial products [24% (95%CI:1–59%)], but lower with lisocabtagene maraleucel [9% (95%CI: 6–13%)], relmacabtagene autoleucel [5% (95%CI: 1–12%)], and tisagenlecleucel [3% (95%CI: 0–8%)] (Figure 9C).

The subgroup analyses of the proportions of patients who received systemic anti-cytokine therapy by study design and costimulatory domain are shown in Supplementary Figures S1, S2.

### Publication Bias

Potential publication bias was assessed. The funnel plot did not show asymmetry. Therefore, there was no evidence of publication bias for the pooled analysis of BOR, BCR, 3-months CRR, 12-months OS, any-grade CRS, severe CRS, any-grade neurotoxicity, and severe neurotoxicity (Supplementary Figures S3A–H).

## Discussion

CAR-T cell immunotherapy demonstrates remarkable achievements in B-cell malignancies. This meta-analysis aimed to summarize and compare current efficacy and safety data of using CAR-T cells targeting CD19 in patients with r/r DLBCL. The present meta-analysis showed a highly favorable clinical response rate in patients with DLBCL treated with anti-CD19 CAR-T cells.

In the present meta-analysis, the pooled 12-months OS rate was 63% (95%CI:56–70%). The subgroup analyses suggested that 4-1BB- [61% (95%CI:47–74%)] and CD28-based products [65% (95%CI:58–71%)] had similar efficacy (*p* = 0.61). Although no statistically significant differences in 12-months OS rates were observed between costimulatory domains, among 4-1BB-based products, relmacabtagene autoleucel appeared to have the highest 12-months OS rate [78% (95%CI:66–88%)], while tisagenlecleucel appeared to have the lowest 12-months OS rate [49% (95%CI:39–58%)]. Significant heterogeneity in the subgroup analyses by different CAR-T products was also observed (I^2^ = 74%, P_heterogeneity_<0.01).

Best response of CAR-T cell therapies varied with different co-stimulatory domains or brands. The result for 1,192 patients in 19 studies showed a BOR estimate of 74% (95%CI: 67–79%) with an estimated 48% (95%CI: 42–54%) BCR among 1,209 patients in 20 studies. Among the included studies, some reported high BCR rates (>60%) (Dean et al., 2019; Jacobson et al., 2020; Nastoupil et al., 2020), while others reported low BCR rates (Kochenderfer et al., 2017; Jacobson et al., 2019; Jaglowski et al., 2019; Locke et al., 2019; Melody et al., 2019; Schuster et al., 2019; Abramson et al., 2020; Ghosh et al., 2020; Iacoboni et al., 2020; Iacoboni et al., 2021; Ying et al., 2021). The reasons for these discrepancies are unknown since the number of studies is too small to determine patterns. Nevertheless, DLBCL is a heterogeneous disease, and patient selection might play an important role in the discrepancies. Different patient populations will have different baseline characteristics. The subgroup analyses suggested that CD28-based CAR-T cell therapies might have a better BOR and BCR than 4-1BB-based therapies. Significant heterogeneity was observed in the pooled results of the non-commercial products since they use different manufacturing technologies, and they included small numbers of patients. To compare with non-commercial products, commercial products have standardized manufacturing construct and stabilizing effect.

Many studies confirmed the high CR rate of CAR-T cell therapies (Lee et al., 2015; Turtle et al., 2016; Locke et al., 2017). However, the higher BCR of CD28 based CAR-T cells did not result in survival benefit than that of 4-1BB based CAR-T cells in the present study. To exclude the impact of time frame, 3-months CRR was analyzed. The pooled 3-months CRR was 41% (95%CI: 35–47%) among 493 patients in six studies. We did not observe the statistically significant differences between 4-1BB [40% (95%CI: 30–51%)] and CD28 [44% (95%CI: 38–49%)] in the subgroup analysis of the costimulatory domain, which is different from the subgroup analyses of the BOR and BCR. This difference could be caused by the inconsistent time frame of the included studies. Hence, the BCR may not be an ideal surrogate outcome of OS to predict the clinical benefit in DLBCL patients. Therefore, it is necessary for future studies to simultaneously report 3-months CRR and 6-months CRR or even 12-months CRR; and then, the ideal endpoint could be determined. Large long-term follow-up studies are necessary to confirm this observation.

CAR-T cell immunotherapy is rapidly developing, but unlike traditional anticancer treatments, they rely on genetically modified cells that require manufacturing processes with a risk of failure (Neelapu et al., 2017; Schuster et al., 2019; Abramson et al., 2020). How to improve efficacy and reduce treatment toxicity remain the most concerning issues. Therefore, CAR design, gene transfection method, cytokine support, expansion and persistence of T cells, preconditioning regimens, infusion dose of T-cells, and types remain to be improved (Feins et al., 2019; Subklewe et al., 2019; Rafiq et al., 2020; Zhang et al., 2020; Sterner and Sterner, 2021). CAR-T cell constructs are complex, and the manufacturing processes will play important roles in the efficacy and safety of the products. After infusion of CAR-Ts, the cells will expand, play their anticancer role, and then go to apoptosis. Thus, the degree of expansion and duration of persistence is often considered to correlate with efficacy (Louis et al., 2011; Porter et al., 2015). The following reasons should be considered when examining CAR-T efficacy. First, a previous study observed that the costimulatory domain could increase persistence (Louis et al., 2011). Indeed, CD28-based CAR-T cells show greater functionality, while 4-1BB-based CAR-T cells display a higher persistence (Kowolik et al., 2006; Brentjens et al., 2007; Plunkett et al., 2007; Milone et al., 2009; Zhong et al., 2010; Santoro et al., 2015; Kawalekar et al., 2016; Guedan et al., 2020; Jafarzadeh et al., 2020). The 12-months PFS rate of 4-1BB products (liso-cel:65%; relma-cel:69.2%; tisa-cel:79%) among patients who had complete response may suggest that longer cell persistence is correlated with better PFS rate, but we didn’t find evidence in CD28 products such as axicabtagene ciloleucel. In addition, lymphodepletion using preconditioning regimens is beneficial to T cell persistence and expansion *in vivo* (Cui et al., 2009; Kohn et al., 2011). All these factors can influence efficacy. Consequently, more attention must be paid to these factors when designing a CAR-T treatment strategy.

Notably, the BOR, BCR and 12-months OS rate of lisocabtagene maraleucel and relmacabtagene autoleucel were numerically higher than those of tisagenlecleucel, even though all the three CAR-T cells were 4-1BB based. CD28 is a transmembrane protein that is constitutively expressed on T cells (Boomer and Green, 2010). 4-1BB is a transmembrane protein that belongs to the tumor necrosis factor receptor superfamily (Pollok et al., 1993). Besides the two costimulatory domains themselves, many pathways are involved in the function and survival of CAR-T cells after infusion (Cappell and Kochenderfer, 2021), leading to different characteristics (Weinkove et al., 2019; Ying et al., 2019; Zhao et al., 2020; Zheng et al., 2020). Indeed, the 4-1BB domain promotes CAR-T survival through the noncanonical NF-κB signaling (Li et al., 2018; Philipson et al., 2020) and tumor necrosis factor receptor-associated factors (TRAFs) (Li et al., 2018). Future studies should examine the influence of these other pathways on the outcomes and whether external factors could be modulated to enhance the efficacy and decrease the toxicity of CAR-T cells.

Nevertheless, the high response rates from CAR-T cell immunotherapy observed in the present analysis come with challenges posed by the adverse events and toxicities. Indeed, in the present analysis, the pooled rates of any-grade CRS, grade ≥3 CRS, any-grade neurotoxicity events, and severe neurotoxicity events were estimated at 78, 6, 41, and 16% of the patients, respectively. Still, the incidence of CRS and neurotoxicity varied greatly among trials. The ZUMA-1 trial reported that any-grade CRS occurred in 93% of the patients, grade ≥3 CRS in 11% of the patients, and severe neurotoxicity in 32% (Locke et al., 2019). The Juliet trial reported 58% of CRS events, 22% of grade 3–4 CRS events, and 12% of grade 3–4 neurotoxicity events (Schuster et al., 2019). The TRANSCEND trial reported that any-grade CRS, grade ≥3 CRS, and grade ≥3 neurotoxicity occurred in 42, 2, and 10% of the patients, respectively (Abramson et al., 2020). Differences in preconditioning regimens, CAR-T cell nature and dose, patient selection, assessment criteria, and sample size will inevitably lead to differences among trials, but the usefulness of a meta-analysis is to pool the results of multiple studies to find patterns. Our analysis suggested that the incidence of any-grade CRS events was higher in anti-CD19 CAR-T cells based on the CD28 costimulatory domain (92%, 95%CI: 89–95%) than with the 4-1BB domain (57%, 95%CI: 47–67%), as well as severe CRS (*p* = 0.04). The difference was also observed for neurotoxicity events (22% for 4-1BB and 64% for CD28) and severe neurotoxicity (*p* < 0.01). These findings suggest that the manufacturing technology might influence CRS and neurotoxicity. In addition, the non-commercial products might have added instability to the analyses. Consistently, the rates of CRS and neurotoxicity were higher with axicabtagene ciloleucel than with relmacabtagene autoleucel, tisagenlecleucel, and lisocabtagene maraleucel. An important factor influencing the clinical outcomes and adverse events is the treatments used to manage the immunological adverse events, including corticosteroids, anti-IL-6 therapy, and anti-seizures drugs (Neelapu et al., 2017; Neelapu et al., 2018). These effects are very complex and study- or practice-dependent. The subgroup analyses of corticosteroid management by costimulatory domain suggested a higher rate of corticosteroid use with CD28 products than with 4-1BB-based CAR-T cells, consistent with the previous safety analyses. A similar tendency was also observed in the subgroup analyses of tocilizumab management by co-stimulatory domain. The influence of such drugs on the prognostic outcomes could not be analyzed in the present meta-analysis.

### Limitations

This meta-analysis has limitations. As for all reviews and meta-analyses, this study inherits the combination of the limitations of the included studies. Therefore, care must be taken when extrapolating and generalizing the results. All patients received CAR-T cells, and no comparison with traditional treatments could be made. The random-effects model was used because heterogeneity among trials was expected due to the differences in design, drugs, doses, outcomes, and methods for measuring the outcomes, while this statistical model cannot completely abolish the actual heterogeneity. The heterogeneity among studies further complicates the comparisons between the reported outcomes in the studies included in the present study, including differences in patient populations, B-cell NHL subtypes disease-specific variables, CAR-T methods, follow-up times, and duration of treatment. Consequently, statistical heterogeneity was observed in many analyses. The CRS and ICANS grading scales used in the included studies were not consistent. Only the two most common but harmful adverse events were considered, but CAR-T cells can have many other safety issues affecting their use, including infections, cytopenia, hypogammaglobulinemia and B-cell aplasia. Finally, the DOR and PFS rates could not be formally analyzed since no available data in CD28-based CAR-T cell studies.

## Conclusion

The present meta-analysis demonstrated excellent effectiveness and manageable safety profile of CD19-targeting CAR-T cells in patients with r/r DLBCL. The subgroup analyses suggested that 4-1BB- and CD28-based CAR-T cells have similar 12-months OS rates and 3-months CRR in patients with r/r DLBCL, but 4-1BB-based CAR-T cells have a better safety profile. Among the 4-1BB products, relmacabtagene autoleucel might have better efficacy than tisagenlecleucel. However, as a newly approved product, relmacabtagene autoleucel lacks real-world data to confirm its long-term clinical benefits. Furthermore, CAR-T cell manufacturing is complex, and discrepancies exist between products using the same costimulatory domain. We suggest future studies to report detailed information about the CAR-T constructs.

## Data Availability

The original contributions presented in the study are included in the article/Supplementary Material, further inquiries can be directed to the corresponding authors.
