# Supplementary figures and images for: Effectiveness and Safety of Anti-CD19 Chimeric Antigen Receptor-T Cell Immunotherapy in Patients With Relapsed/Refractory Large B-Cell Lymphoma: A Systematic Review and Meta-Analysis
Source: Front Pharmacol. 2022 Apr 25;13:834113. doi: 10.3389/fphar.2022.834113 (PMC9081610; doi:10.3389/fphar.2022.834113)

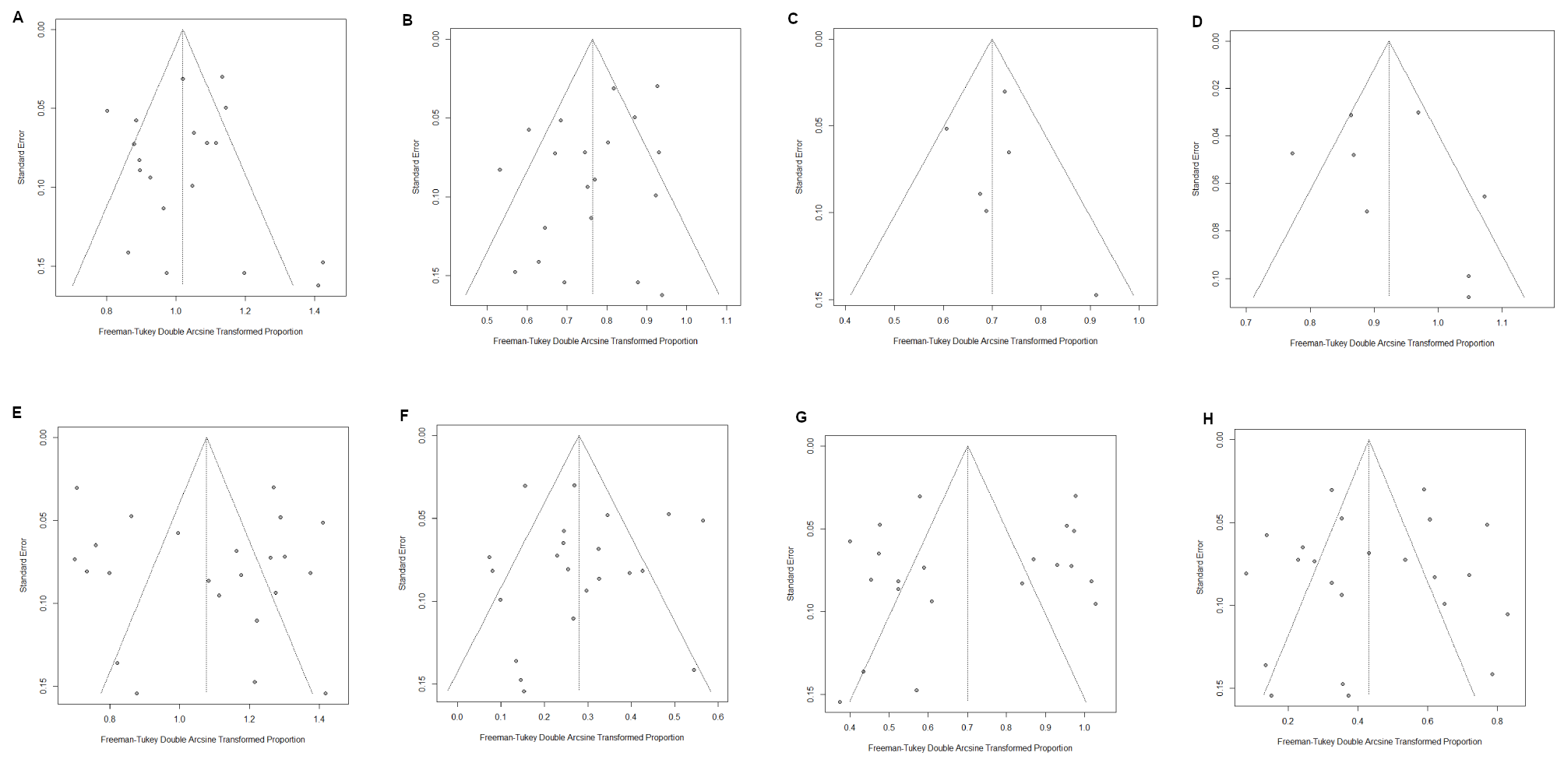

Supplement: Supplementary file 1 [file Image3.tiff]

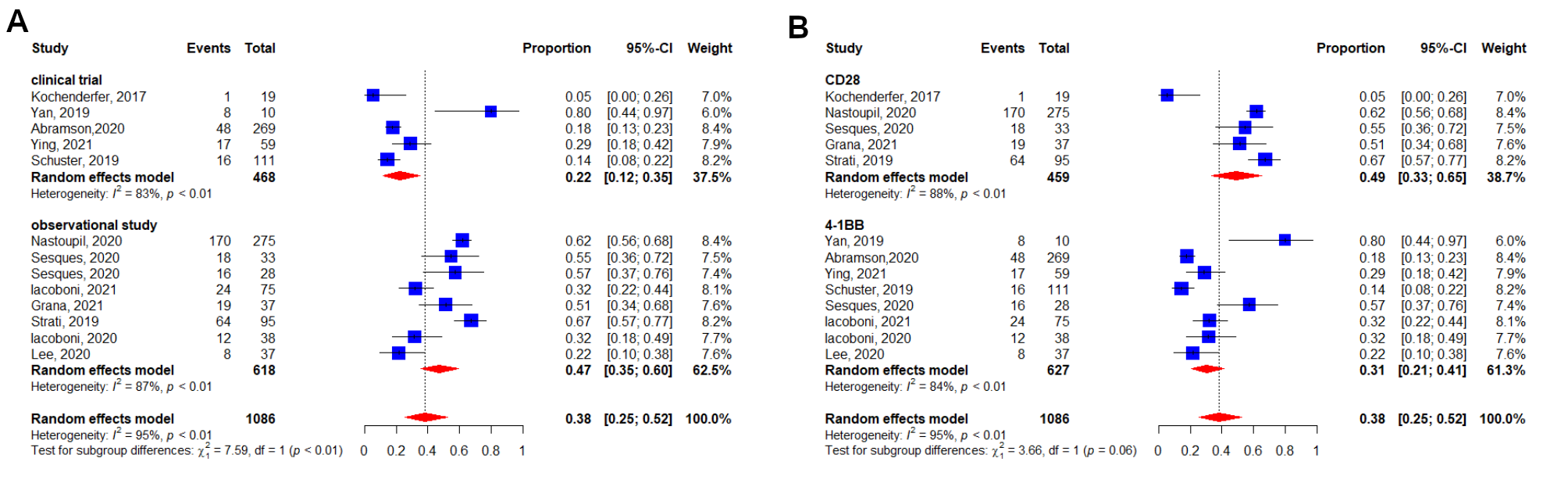

Supplement: Supplementary file 2 [file Image1.tiff]

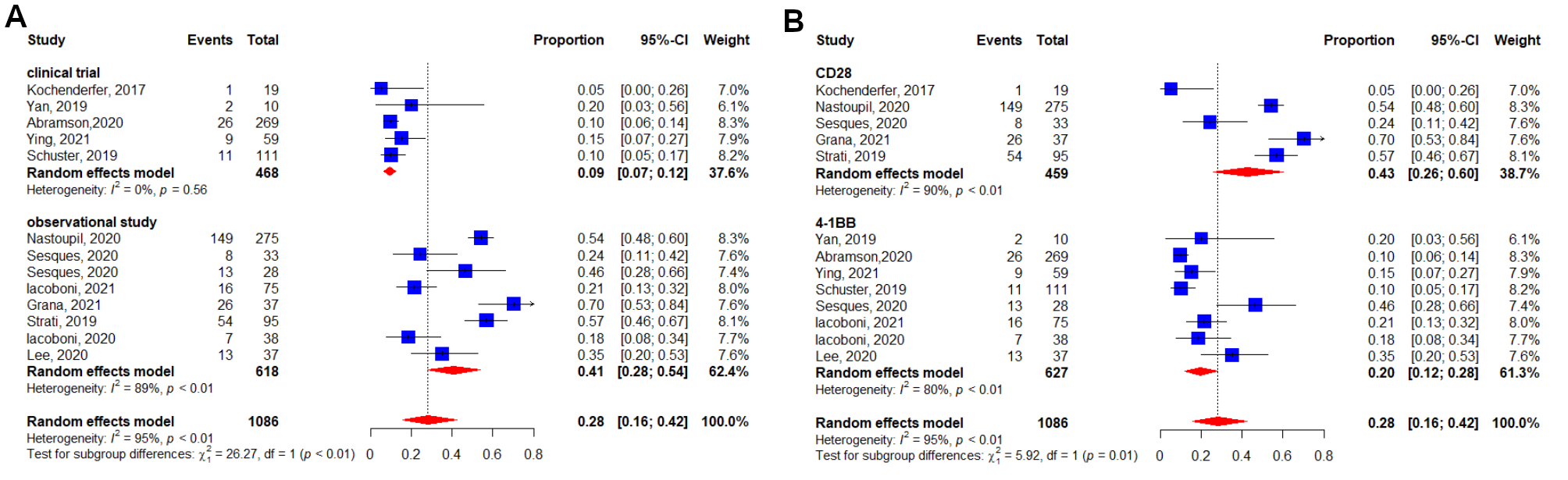

Supplement: Supplementary file 5 [file Image2.tiff]
