# Supplementary material for: Effectiveness and Safety of Anti-CD19 Chimeric Antigen Receptor-T Cell Immunotherapy in Patients With Relapsed/Refractory Large B-Cell Lymphoma: A Systematic Review and Meta-Analysis
Source: Front Pharmacol. 2022 Apr 25;13:834113. doi: 10.3389/fphar.2022.834113 (PMC9081610; doi:10.3389/fphar.2022.834113)
Supplement: Supplementary file 3 [file Table1.docx]

**Supplementary Table S1.** NOS criteria for the observational studies

| Study | Representativeness of the exposed cohort | Selection of the non exposed cohort | Ascertainment of exposure | Demonstration that outcome of interest was not present at the start of the study | Comparability of cohorts based on the design or analysis | Assessment of outcome | Was follow-up long enough for outcomes to occur | Adequacy of follow up of cohorts | Total quality scores |
| --- | --- | --- | --- | --- | --- | --- | --- | --- | --- |
| Nastoupil, 2020 [30] | ★ | ★ | ★ | / | ★ | ★ | ★ | ★ | 7 |
| Sesques, 2020 [70] | ★ | ★ | ★ | / | ★ | ★ | / | ★ | 6 |
| Iacoboni, 2021 [27] | ★ | ★ | ★ | ★ | ★ | ★ | ★ | ★ | 8 |
| Grana, 2021 [31] | ★ | ★ | ★ | / | / | ★ | / | ★ | 5 |
| Pennisi, 2020 [74] | ★ | ★ | ★ | / | / | ★ | / | ★ | 5 |

**Supplementary Table S2**. MINORS instrument for clinical trials

| Study | A clear stated aim | Inclusion of consecutive patients | Prospective collection of data | Endpoints appropriate to the aim of the study | Unbiased assessment of the study endpoint | Follow-up period appropriate to the aim of the study | Loss to follow-up less than 5% | Prospetive calculation of the study size | An adequate control group | Contemporary groups | Baseline equivalence of groups | Adequate statistical analyses | Total |
| --- | --- | --- | --- | --- | --- | --- | --- | --- | --- | --- | --- | --- | --- |
| Locke, 2019 [28] | 2 | 2 | 2 | 2 | 2 | 2 | 2 | 1 | 2 | 2 | 2 | 2 | 23 |
| Huang, 2020 [66] | 2 | 0 | 2 | 2 | 2 | 1 | 2 | 0 | 2 | 2 | 2 | 2 | 19 |
| Kochenderfer, 2017 [36] | 2 | 0 | 2 | 2 | 2 | 2 | 2 | 0 | 2 | 2 | 2 | 2 | 20 |
| Yan, 2019 [67] | 2 | 2 | 2 | 2 | 2 | 1 | 0 | 0 | 2 | 2 | 2 | 2 | 19 |
| Zhou, 2020 [26] | 2 | 2 | 2 | 2 | 2 | 2 | 2 | 0 | 2 | 2 | 2 | 2 | 22 |
| Abramson, 2020 [24] | 2 | 2 | 2 | 2 | 2 | 2 | 2 | 0 | 2 | 2 | 2 | 2 | 22 |
| Ying, 2021 [29] | 2 | 2 | 2 | 2 | 2 | 1 | 2 | 2 | 2 | 2 | 2 | 2 | 23 |
| Schuster, 2019 [25] | 2 | 2 | 2 | 2 | 2 | 2 | 2 | 2 | 2 | 2 | 2 | 2 | 24 |
| Not reported | 0 | 2 | 0 | 0 | 0 | 0 | 1 | 5 | 0 | 0 | 0 | 0 | 8 |
| Reported inadequate | 0 | 0 | 0 | 0 | 0 | 3 | 0 | 1 | 0 | 0 | 0 | 0 | 4 |
| Reported adequate | 8 | 6 | 8 | 8 | 8 | 5 | 7 | 2 | 8 | 8 | 8 | 8 | 84 |
